# ‘You’re setting a lot of people up for failure’: what formerly incarcerated women would tell healthcare decision makers

**DOI:** 10.1186/s40352-022-00166-w

**Published:** 2022-02-01

**Authors:** Whitney K. Norris, M. Kathryn Allison, Marley F. Fradley, Melissa J. Zielinski

**Affiliations:** 1 University of Arkansas for Medical Sciences, Little Rock, AR USA; 2 University of Arkansas, Fayetteville, AR USA

**Keywords:** Women, Prison, Reentry, Health care, Criminal justice system

## Abstract

**Background:**

Incarcerated women have a higher prevalence of health problems than the general population; however, little is known about their perspectives on the healthcare they receive. Here, we conducted semi-structured interviews with women who had been incarcerated (*n* = 63) which asked what they would tell healthcare decision-makers about their experiences of healthcare in prisons and the community post-incarceration if provided the opportunity. All participants had a history of sexual violence victimization and had at least one period of incarceration in a community corrections center in Arkansas due to the goals of the larger study from which data were drawn.

**Results:**

Four themes arose when participants were asked what they would tell people who make decisions about community healthcare: 1) the healthcare system is not working (52%; *n* = 33), 2) have compassion for us (27%; *n* = 17), 3) recognize that we have specific and unique needs (17%; *n* = 11), and 4) the transition from incarceration is challenging and requires more support (22%; *n* = 14). Three themes arose when we asked participants what they would tell people who make decisions about healthcare in prisons: 1) we had experiences of poor physical healthcare in prison (44%; *n* = 28), 2) more specialty care is needed in prison (49%; *n* = 31), and 3) healthcare providers treat women in prison poorly (37%; *n* = 23).

**Conclusions:**

Our findings underscore the need for systemic changes including greater oversight of prison-based healthcare services, enhanced access to medical subspecialties in prisons, and healthcare provider training on the unique needs of incarcerated and previously incarcerated women. Polices that expand healthcare access are also likely to benefit formerly incarcerated women given the challenges they experience seeking community-based care.

## Introduction

Between 1980 and 2019, the United States (U.S.) saw a 665% increase in the number of incarcerated women, more than double the pace of growth among men (Carson, 2020; Minor-Harper, 1982; Sawyer, 2018). Incarcerated women’s physical and mental health conditions and needs differ significantly from incarcerated men and from women in the general population. For example, incarcerated women have a higher prevalence of childhood trauma (Hayes, 2015; Roos et al., 2016); sexual victimization (Bucerius et al., 2021; DeHart, 2008; Karlsson & Zielinski, 2020); mental illness, including addiction (Karlsson & Zielinski, 2020; Fazel et al., 2017); sexually-transmitted diseases (Nijhawan et al., 2010); and many chronic physical health conditions, including hypertension, diabetes, myocardial infarction, asthma, arthritis, cervical cancer, and hepatitis (Binswanger et al., 2009; Knittel et al., 2021).

Women also have gender-specific needs. For example, most incarcerated women are of childbearing age and about 4% enter prison pregnant and in need of obstetrical care (Sufrin et al., 2019). One of the most significant gender differences is the high prevalence of sexual assault—often referenced as “the pathway to prison”—experienced by women involved in the criminal justice system (DeHart, 2008; Karlsson & Zielinski, 2020). A recent review found that incarcerated women have a lifetime prevalence of sexual violence victimization of 56%–82% (50%–66% in childhood and 28%–68% in adulthood; Karlsson & Zielinski, 2020), which is at least three times higher than the rate among incarcerated men (Clark et al., 2012; Lane & Fox, 2013; Komarovskaya et al., 2011). Victims of childhood sexual abuse experience significantly more health complaints than comparison groups (see Irish et al., 2010 for a meta-analysis) and sexual violence victimization more generally has demonstrated robust associations with the development of mental illness (Dworkin, 2018; Dworkin et al., 2017).

These findings point to the need for high quality physical and mental healthcare within prisons and the need to attend to women’s healthcare access within communities after being released from prison. Here, we interviewed formerly incarcerated women about their healthcare experiences and asked them what they would tell those who make decisions about healthcare in prisons broadly and in the community about their health and healthcare needs. All participants had a history of sexual violence victimization and were previously incarcerated in a specific community corrections center in Northwest Arkansas due to the goals of the larger study from which data were drawn.

### Healthcare in prison

People who are incarcerated have a constitutional right to healthcare that is of comparable quality to that in the community. Prison may provide a unique opportunity to get people who are disconnected from healthcare back into treatment or into treatment for the first time (Besney et al., 2018; Nijhawan et al., 2010). However, numerous studies found that incarcerated people report that access to prison-based health care is limited and quality is poor (Alves et al., 2016; Barry et al., 2019; Young, 2000), with women reporting that they are being treated as “sub-human” by the system (Harner & Riley, 2013). Those serving longer sentences in the Harner and Riley (2013) study specifically voiced that this trend of poor treatment by correctional officers seems to be worsening. In Young’s (2000) qualitative study of women’s perception of healthcare in prison, all participants mentioned at least one example of unempathetic care from a prison healthcare provider and reported significant delays in receiving needed care. Conversely, some studies comparing health outcomes of people who are incarcerated to similar samples in the community found improved health outcomes—likely due to extremely poor quality healthcare access/engagement in the community (e.g., healthcare for incarcerated pregnant women; Baker, 2019).

Recent nationally representative studies of healthcare in women’s prisons are lacking. A national survey of women’s prisons conducted in 2001 found that while the facilities surveyed met the basic healthcare needs of incarcerated women, the availability of specialty services for chronic disease, disability, and mental health—conditions more prevalent among this population—was less consistent (Young & Reviere, 2001). A 2016 qualitative study in Canada also revealed significant barriers to prison healthcare intake procedures, including a lack of knowledge about what services are offered as well as significant issues with efficiency, confidentiality, and communication surrounding the Health Service Request form process (Ahmed et al., 2016). These incarcerated women made specific recommendations including a need for more comprehensive entrance and exit health assessments, more health literacy education, and the need for health support networks (Ahmed et al., 2016). To the authors’ knowledge, researchers have not conducted similar studies in U.S. prisons and thus the applicability of these results to other prison systems is unknown, presenting a gap in our current knowledge.

### Post-incarceration healthcare

Due to much higher morbidity rates immediately upon release (Binswanger et al., 2007), most research on post-incarceration healthcare in countries similar to the U.S. (e.g., Canada) focuses specifically on the short-term or immediate transition from prison to the community (Colbert et al., 2016, 2013; Fox et al., 2014; Shavit et al., 2017). Women in Ahmed et al.’s (2016) study in Canada spoke of “fragmentation” or interruption of healthcare upon entry and release from prison as a barrier to healthcare, including services such as continuity of pharmaceutical care for chronic conditions and mental health diagnoses. They also described how experiences with the prison healthcare system negatively impacted their post-release healthcare engagement, including provoking feelings of disempowerment around accessing healthcare. Similarly, women in Abbott et al.’s (2017) study of justice-involved women in Australia, which involved both pre- and post-release interviews about their healthcare experiences, found that women described their healthcare access as a kind of “medical homelessness” marked by transient and disrupted care. Women also described stigma and a fear of stigma as barriers to their community healthcare engagement.

Other studies of post-release healthcare used all-male samples and/or focused on specialty healthcare, such as treatments for HIV, hypertension, opioid dependence, and diabetes (Fox et al., 2014), healthcare related to drug use behaviors and/or treatments (Calcaterra et al., 2014; Chamberlain et al., 2019; Johnson et al., 2013), stigma as a barrier to access (LeBel, 2012; van Olphen et al., 2009), and healthcare access prior to incarceration (Oser et al., 2016). Post-incarceration studies in male samples found financial and administrative barriers to accessing healthcare in the community (Marlow et al., 2010; Vail et al., 2017). However, little is known about women’s experiences of accessing healthcare in the community longer-term post-incarceration, particularly in the U.S.

### The current study

Incarcerated and formerly incarcerated women have unique and significant healthcare needs, including care that addresses both physical and mental health conditions. However, most research in this area centers around either questions chosen by researchers, specific conditions or programs (such as substance use, Staton et al., 2003; or aging, Reviere & Young, 2004), or focuses only on healthcare in prison or the immediate/short-term post-release period. As a result, incarcerated and formerly incarcerated women’s voices and longer-term perspectives regarding health and healthcare priorities and policy are lacking. The goal of this study was to elevate formerly incarcerated women’s voices regarding their perspectives and opinions about healthcare in prison across the facilities they have been sentenced to and in the community 3-5 years after their release using semi-structured qualitative interviews.

## Methods

### Participants

Due to the goals of the larger study from which this data came, potential participants were women who 1) had been incarcerated at a residential corrections center in Northwest Arkansas at least once between January 2012 and May 2017 and 2) had a history of sexual violence victimization.^1^ We selected these dates so that women recruited for the study would be at least three years post-incarceration by the time of their participation. This fit with our goal of better understanding women’s longer-term needs and perspectives and differentiates our work from studies focusing on the immediate post-release period. Eligibility requirements included: being 18 years old or older, ability to speak English, ability to give consent, history of incarceration at Northwest Arkansas Community Corrections Center^2^ during the target dates, history of sexual trauma, and willingness to allow the research team to confirm incarceration dates using administrative data.

Participants were 63 women with an average age of 37. Ten participants (15.9%) were re-incarcerated at the time of the interview. All participants had had multiple jail stays and most had multiple prison stays (*n* = 37; 58.7%) from which they could draw when answering the interview questions; the median number of times participants reported spending more than 1 day in jail was 7 (*M* = 8.93, range = 2–40) and the median number of separate prison stays was 2 (*M* = 2.34, range = 1–6).

Consistent with the demographics of the facility population where we focused our recruitment efforts, most participants were White (*n* = 52, 82.5%). Approximately half of participants (*n* = 37, 58.7%) reported attending at least some college. See Table 1 for more demographic information.

**Table 1:** Participant Demographics

	*Total % (N)*
*Age*	
18–29	15.9% (10)
30–39	49.2% (31)
40–49	23.8% (15)
50–59	11.1% (7)
*Race*	
American Indian/Alaskan Native	6.3% (4)
Asian	1.6% (1)
Black or African American	1.6% (1)
White	82.5% (52)
More than one race	4.8% (3)
Unknown	3.2% (2)
*Ethnicity*	
Hispanic or Latino	9.5% (6)
Not Hispanic or Latino	90.5% (57)
*School completed*	
Some high school	6.7% (4)
Graduate equivalent (GED)	19.0% (12)
High school graduate	12.7% (8)
Some college	52.4% (33)
College graduate	6.3% (4)
Unknown	3.2% (2)
*Incarceration status at time of interview*	
Re-incarcerated	15.9% (10)
In the community	84.1% (53)

### Procedure

To facilitate recruitment for the larger mixed methods study, the Arkansas Division of Community Corrections provided the researchers with a list of women who were incarcerated at a residential community correction center between January 2012 and May 2017. We then used a combination of purposive sampling and snowball sampling to recruit participants between May 2019 and November 2020. We primarily relied on publicly available contact information (e.g., online directories and social media) to reach people who were likely to qualify for the study and provide consent to contact. We also placed recruitment flyers at targeted locations (e.g., AA meetings and probation offices in areas of the state where women most often paroled). A member of the research team then contacted potential participants by phone, email, or social media messaging and asked them to complete an eligibility screening. Each potential participant completed a brief screening in-person or over the phone to determine eligibility.

After obtaining consent, we gave eligible participants the option to schedule an interview in-person or over the phone. Semi-structured interviews averaged approximately 50 minutes. We reimbursed participants between $30–$50 for their time. We audio recorded all interviews and transcribed them verbatim. Once checked for accuracy, our research team analyzed all text data using MAXQDA qualitative software. Data used for this analysis consisted of responses to the two following questions, which were a part of the larger participant interview:

1. If you had a chance to tell the people who make decisions about healthcare in our communities anything you want to about formerly incarcerated women’s physical and mental health care needs, what would you tell them?
2. If you had a chance to tell the people who make decisions about healthcare in prisons anything you want to about formerly incarcerated women’s physical and mental health care needs, what would you tell them?

Two members of the research team (the first and second authors) performed a thematic analysis of the transcribed interview data based on descriptive qualitative analysis beginning with open coding (Sundler et al., 2019). After development of the initial codebook, the primary investigator of the parent study (the last author) provided feedback and the research team made alterations to the codebook collaboratively during group discussion. Coding was iterative in nature with each member of the research team coding the data independently and meeting to discuss areas of agreement and disagreement in coding. Once the codebook was agreed upon by all parties, the three coders reviewed coding of all transcripts to ensure consistency.

## Results

Four main themes arose when participants were asked what they would tell people who make decisions about community healthcare regarding formerly incarcerated women’s physical and mental healthcare needs. Three main themes arose when we asked participants what they would tell people who make decisions about healthcare in prisons about incarcerated women’s physical and mental healthcare needs. These results are divided into two sections below: healthcare in communities and healthcare in prison. Table 2 also provides a brief summary of all themes and contributing subthemes.

**Table 2:** Formerly Incarcerated Women’s Messages for Healthcare Decision Makers (Themes and Sub-Codes)

Community Healthcare	Prison Healthcare
**Theme 1: The healthcare system is not working.** 1. Insurance is a barrier 2. Cost is a barrier 3. Lack of access leads to more poor health outcomes 4. Mental health care is not available 5. Stigma and limits placed on formerly incarcerated women are barriers	**Theme 1: We had experiences of poor physical healthcare in prison.** 1. Stories of poor physical healthcare experiences 2. Behavioral healthcare experiences were positive (programs, groups, counselors) 3. Generally satisfied/neutral experiences of physical healthcare
**Theme 2: Have compassion for us.** 1. We matter 2. Listen to us 3. We need more help/resources, not punishment	**Theme 2: More specialty care is needed in prison.** 1. Need for more/better physical healthcare options/services a. Dental & eye care b. Better chronic illness care c. Better screenings/assessments 2. Need for more/better mental healthcare a. Addiction-specific b. Trauma-specific c. More individualized care (including more frequent sessions) d. Better mental health screening at intake
**Theme 3: Recognize that we have specific and unique needs.** 1. Need for recognition of unique needs of formerly incarcerated women 2. Mental health is especially important, especially in light of trauma histories 3. Need for understanding of trauma and addiction	
**Theme 4: The transition from incarceration is challenging and requires more support.** 1. Women are returning to same environments/lack support 2. Expectations are unrealistic 3. Finding employment is difficult 4. Need for more formal support (transitional living, social workers, etc.)	**Theme 3: Healthcare providers treat women in prison poorly.** 1. Need for providers to believe, listen to, and take seriously the needs of incarcerated women 2. Need for empathetic, nonjudgmental providers who are there because they are care 3. Need to be treated like a human being

### Healthcare in communities

When asked what they wanted to share about healthcare in communities, participants shared about how the healthcare system is not working for formerly incarcerated women, due to issues of access and availability of care (52%; *n* = 33). They also described a need for compassionate providers (27%; *n* = 17) and a need for providers who recognize the specific and unique healthcare needs of this population (17%; *n* = 11). The challenges of the transitional period from prison to the community also arose (22%; *n* = 14).^3^

#### The healthcare system is not working

Many participants spoke about access issues. Insurance, specifically, was the most commonly mentioned barrier to needed healthcare services due to issues such as lack of coverage availability, “restrictions,” and requirements. Cost was also mentioned as a barrier. One participant stressed that “a lot more people would get the help they needed if they could afford it.” Others spoke to the general lack of affordability of healthcare services even when covered by insurance due to issues such as high co-pays and “additional charges,” lack of adequate coverage for and availability of needed services (especially mental health services), and the need to prioritize other monetary demands such as court fines. One participant requested that decision makers “take into consideration everything that someone who’s come out of incarceration has to pay” and spoke to the competing needs of working enough to pay fines while not working more than the maximum hours to qualify for her healthcare. Another participant linked this theme to recidivism by stating:

“If you’re poor … you are not going to get the care that you would have if you weren’t poor … We don’t get that. So, we end up dying or getting strung out on drugs or being put in mental institutions, committing crimes to go back to prison.”

#### Have compassion for us

Many participants called for more compassionate treatment for incarcerated women within the healthcare system broadly. One participant stated, “Think of it as if it might be your child, or maybe if it was your mom, or maybe it was you. How would you want to be treated?” Another woman expressed that compassionate treatment “makes all the difference.” Acknowledging stigma associated with having been incarcerated, participants expressed the desire to be listened to, not to be “judged,” and to be treated like they matter—“like we’re somebody”—regardless of their past choices. One woman called the “stigma” and “condemnation” of people who have been incarcerated “counterproductive” and said, “all that toxic shame, it’s not getting people anywhere.”

A sub-theme also emerged in which participants spoke to the need for more help and resources instead of punishment. One participant summed this up well by stating:

“If you’re dealing with a kid who’s acting up... the only reason they’re doing it is because something is wrong. That’s the same reason why adults do drugs or just things like that. And if they would address the issue instead of just locking them up, … the world would be a better place … Because if you know better, you tend to do better … Instead of building so many prisons, why not build rehabs or make people go to places like [local rehabilitative community corrections center] instead of just throwing them in prison.”

#### Recognize that we have specific and unique needs

Many women spoke to a need to not only be treated like human beings without the stigma of having been formerly incarcerated, but also the need for those involved in healthcare to understand that formerly incarcerated women have specific and unique health needs due to the history that likely led to their involvement in the criminal justice system, including histories of trauma and addiction. One woman simply said, “We’re dealing with a lot of stuff that we don’t talk about … we hold a lot of stuff in.” Another requested that decision makers and providers “look at the big picture and not to just … treat us like anybody.” One participant spoke specifically to the role of trauma in this regard:

“The center of all these things that are going on in these women’s lives is the trauma. Trauma healing is where it is at. If they can get that they can overcome so many more obstacles and that’s the most important … everybody’s a unique individual, and different ways help different people, but I think the source of all these issues are definitely trauma.”

#### The transition from incarceration is challenging and requires more support

Many participants spoke about the difficulties involved in the transition from prison to the community and gave examples of specific resources needed during this transition. One participant described “culture shock” when she was first released and spoke to the expectations being “unrealistic.” Many participants spoke to the specific struggles of this transition period, including returning to unhealthy or unsafe environments, financial instability, and issues finding and maintaining employment. For example, one participant said, “Right when you get out, you don’t have anything.” Others suggested that women need more formal support during the transition out of incarceration, such as transitional living or assigned social workers. In describing the high demands of the post-transition period, one participant stated:

“They’re trying to get any job they can get. On top of maintaining any meetings they were required to do by the court, on top of seeing your probation and parole officers. If you have kids, you’re like, ‘This is just a lot.’ You’re setting a lot of people up for failure at this point … Trying to figure out how to balance all of it. That’s a lot. That’s just too much for one person.”

### Healthcare in prisons

When asked what they wanted to share about healthcare in prisons, participants shared about (1) poor physical healthcare experiences (44%; *n* = 28), (2) a lack of needed specialty care (49%; *n* = 31), and (3) being treated poorly by healthcare providers in prison (37%; *n* = 23).^3^

#### We had experiences of poor physical healthcare in prison

When asked what they would tell decision makers about healthcare in prison, a few participants said they were satisfied overall with the physical and/or mental healthcare they received; however, many more participants shared stories about their poor experiences with physical healthcare. One participant simply answered by saying, “It needs to improve tremendously.” Another participant reported she had a severe ear infection for her first thirty days of incarceration and nothing was done about it. She said, “It’s got to be the worst healthcare that I have ever ran into. Weeks to get in whenever something’s terribly wrong with you.”

Women talked about major delays in receiving healthcare when needed or requested, as well as the negative health outcomes that resulted from these unnecessary delays. One participant reported that “it was really hard to get in” and that you were required to pay for two visits to the nurse before seeing the doctor for something as minor as “a sinus infection or something.” Another participant told a story about having a kidney infection while incarcerated. She explained, “It took them 48 hours to get to me. They thought I was faking it … I had a temperature of like 105 degrees before they did something.”

#### More specialty care is needed in prison

A majority of participants expanded on specific needs regarding both physical and mental healthcare services that were not adequately provided to them in prison. Some spoke to the need for more mental health services, including services specific to trauma and addiction, and the need for more individualized mental health treatment. One participant said, “It’d be nice to have more counselors so that they could see less clients and see us more often than once a month.” Women also spoke to the need for more comprehensive screenings at intake and a need for better chronic illness care. One participant explained, “The chronic care I think needs to be not taken so lightly.” The need for and lack of dental care services outside of tooth extractions also came up several times during the interviews: “If you get a cavity, they don’t even bother filling it. They just pull out your tooth.” Many women made the point that prison is an opportune time for this population to receive treatment for unmet physical and mental health needs. One participant said, “I think that they should offer a lot more services. We’re supposedly state property. Well, if we’re state property, fix us.”

#### Healthcare providers treat women in prison poorly

Many women brought up experiences of being treated poorly by healthcare providers in prison. One participant simply explained, “It would be nice to be treated like a human in prison by a doctor.” Many participants talked about the need for empathetic, non-judgmental providers who choose to work in the prison system because they care about incarcerated people. One participant said, “They don’t care about our wellbeing enough. They give you the bare minimum of what they can to take care of you while you’re under their charge.”

Not being listened to or taken seriously by prison providers was a common sub-theme. One formerly incarcerated woman summarized what she would tell decision makers about her experience of healthcare in prison by saying:

“It feels like it’s ran by cold, callous, uncaring people who don’t care if you live or die at all. It feels like the ones who do care can’t do anything about it. The ones that are in charge don’t care if you’re alive or dead...And it’s terrifying if you’re in there … what are you going to do? Who are you going to complain to? It’s terrifying. And no one’s going to … you’re helpless.”

## Discussion

Justice-involved women are a population of high healthcare need (Binswanger et al., 2009). In this study, we sought to learn more about formerly incarcerated women’s perspectives on healthcare in prison and healthcare in the community after prison. Participants provided rich insights based on their experiences. We learned that these women have specific physical and mental healthcare needs that are often not addressed in prison or in the community due to a variety of obstacles, including lack of access, lack of adequate support, and lack of individualized, humane, and compassionate care. Emergent themes provide direction for policymakers and healthcare providers to improve the healthcare experiences and access of formerly incarcerated women and as a result possibly reduce recidivism and a variety of negative health outcomes. A deeper dive into each of the themes that emerged may prove beneficial to inform future healthcare policies.

We found it notable that many participants—when given the opportunity to say *anything* that they wished to say to healthcare decision makers—told us detailed stories about having experienced or witnessed poor treatment by prison healthcare providers. While these results were not surprising (c.f., Colbert et al., 2013; Young, 2000), the frequency at which we heard these narratives may speak to the salience of these experiences, even several years after release from incarceration. While we did not ask and therefore cannot speak to the impact that these healthcare experiences may have had on future healthcare engagement, it is conceivable that negative experiences could dissuade future service access, as has been found in related samples (e.g., people who use or inject drugs; Biancarelli et al., 2019; Muncan et al., 2020). Moreover, the gaps in foundational healthcare that women voiced (e.g., lack of dental services, evidence-based therapy) were numerous and linked with the kinds of poor health outcomes often evidenced post-release.

Somewhat surprisingly given that all women in our study sample had experienced sexual assault, there was little discussion of healthcare needs related to sexual assault specifically. It could be that women were hesitant to share about these needs or that they were not top of mind when asked what they would share with healthcare decision makers if given the opportunity to say *anything*. It is also possible that our sample did not see their sexual assault status as clearly connected to their current healthcare needs. Because we did not probe for sexual assault care needs specifically, we cannot know. However, existing literature already demonstrates that these needs do exist in prisons and that there is a need for healthcare, including therapy, that are tailored to these experiences. For example, SHARE is a promising therapy for women survivors of sexual assault who are currently incarcerated (Zielinski et al., 2021). However, more research is needed on how to best support the recovery of sexual assault survivors who become incarcerated to inform healthcare priorities.

Our results aligned with previous literature on the need for specialty care in prisons, including gender-specific care (Besney et al., 2018) and mental health care (Fuentes, 2014). Previous research has specifically pointed to the need for mental health treatment, especially trauma-specific treatment, to decrease recidivism in criminal justice-involved women (Fuentes, 2014). Much like the participants in the current study, others have called for the necessity of knowledge of trauma-informed care for professionals working with incarcerated women (Harner & Burgess, 2011). In addition, other researchers have pointed to the importance of specialized addiction services in prisons due to the higher prevalence of substance abuse and dependence in these individuals than the general population (Fazel et al., 2006).

Women’s experiences with post-release healthcare included more prescriptive interpersonal themes than their generally more descriptive, story-oriented responses to questions about healthcare in prisons. In early versions of the codebook, we had the data connected to “Have compassion for us” and “Recognize that we have specific and unique needs” as a single theme. As we continued working with the data, we realized that most of these women were not only calling for compassionate treatment that should be given to any human being, but they would also tell healthcare decision makers that their experiences and needs are different and perhaps more complex than those of women who have never experienced incarceration. Several women explained that this consideration would not only improve their health and quality of life but that empathetic attitudes and understanding from providers and decision makers could also reduce recidivism. The participants’ greater emphasis on the interpersonal patient-provider dynamics of community healthcare may reflect that they felt more in control of their healthcare decisions and more able to access basic needed services in the community—in comparison to the limited power and options available under correctional control—making provider quality and fit more central elements of their healthcare experiences.

These characteristics may also be particularly salient with interpersonal violence survivors (Reeves & Humphreys, 2018) and highlights the value of arming healthcare providers with training in trauma-informed care. Given the sheer volume of people who experience incarceration in the U.S., we would also recommend that training programs for healthcare providers provide education about mass incarceration, its impacts, and its intersection with trauma and adversity. Without a concerted effort to educate providers, it is likely that people returning to the community from incarceration will continue to see providers that lack the training to fully appreciate their experiences and anticipate their needs. Our study also supports the role of models such as that used by the Transitions Clinic Network in which community health workers with a history of incarceration are embedded in clinics to support people seeking care shortly after release from incarceration (Shavit et al., 2017; Wang et al., 2010).

The largest unique contribution of this study is the fact that it gives us insight into formerly incarcerated women’s healthcare needs and perspectives 3-5 years post-incarceration in a community corrections center. Previous research in this area only provides information on healthcare immediately following release, which the women in this study explained is an especially difficult time and may not be representative of formerly incarcerated women’s perspectives on healthcare. Indeed, reentry may be a time when it is challenging to prioritize healthcare given all the challenges that women face during this period. Many women spoke specifically of how overwhelming the transition experience was and how the expectations are unrealistic, especially considering the many legal, financial, and family obligations present immediately following release. Several women provided specific solutions, such as more transitional living options and the availability of case managers and peer support specialists. Research is needed to understand how to make these services more accessible and effective given intersecting challenges such as parole requirements that disallow cohabitation among people with prior felony convictions, lack of funding, and poor reimbursement. Better understanding of how existing services have been funded and sustained may be particularly valuable.

A strength of our study is the relatively large sample size (*N* = 63). The nature of the open-ended questions also enabled us to learn more about participants’ views as opposed to questions only focused on specific health problems or programs. However, our study was not without limitations. Though our study sample matched the demographic make-up of the community corrections center population involved in the study, this sample lacked racial and socioeconomic diversity as compared to other prison samples which likely impacted the themes we found as well as the overall generalizability of these results to the experiences of all incarcerated women. With a more diverse sample, we may have learned more about experiences of discrimination in healthcare at the intersection of history of incarceration and minority status. Although some women drew upon the experiences of multiple facilities in their answers, the sample was drawn from a community corrections center that specializes in rehabilitative services, which may have impacted the perspectives of these women compared to other potential prison samples. The sample for this study was selected from a parent study that had additional sample restrictions (i.e., history of sexual assault) and may not be generalizable to all incarcerated women. Since this population has such a high prevalence of trauma and sexual violence (Karlsson & Zielinski, 2020), we do not believe these characteristics will differ significantly from the broader population of women who serve sentences in community corrections centers. However, future research on the healthcare priorities of formerly incarcerated women should aim to engage a more diverse sample which also includes participants outside of the community corrections center/rehabilitation-focused prison population.

## Conclusions

Our findings illustrate significant barriers to accessibility and availability of healthcare services for formerly incarcerated women, as well as needed improvements regarding healthcare in prison and in the community after prison. In addition to confirming some of what we already know about the poor healthcare access and experiences of this population, this study highlighted several possible points of intervention in policy and practice. Women transitioning out of prison need programs and services that provide more support and follow-up as well as policies that account for the burdens placed on women during the transition from prison. These needs have been previously articulated within the Bangkok Rules, a set of international standards for the treatment of justice-involved women adopted in 2010, as well as guidelines on reentry for women (Substance Abuse and Mental Health Services Administration, 2020) and people with behavioral health disorders (Substance Abuse and Mental Health Services Administration, 2017). The degree of implementation and effectiveness in routine practice has, to the authors’ knowledge, not been studied. Additionally, better insurance options are needed for women with lower incomes and for those who struggle to find stable employment after incarceration. However, providing better insurance options is only one part of the equation (c.f., Howell et al., 2019). This study also found that a lack of healthcare literacy—a foundational understanding of basic health information needed to make informed decisions about healthcare—was also a significant barrier to accessing quality care. Even if better insurance and healthcare options are provided to this population, many women will still require education around how to access or utilize these services. Additional critical systemic changes include greater oversight of prison-based healthcare services, enhanced access to medical subspecialties in prisons, and integrating training on the unique needs of incarcerated and previously incarcerated women in educational curricula for healthcare providers. Policymakers and healthcare providers need to increase their understanding of what we know about the experiences and needs of this population, especially in regard to their higher prevalence of traumatic stress and need for adequate physical and mental healthcare which they may need significant help accessing. Polices that expand healthcare access are also likely to benefit formerly incarcerated women given the challenges they experience seeking community-based care (Dickson et al., 2018).

## Data Availability

Not applicable.

## Footnotes

1 The larger study required that women have a history of sexual violence victimization because one goal of the project was to compare post-release outcomes of women who had and had not completed an intervention for sexual assault recovery while incarcerated. This intervention, Survivors Healing from Abuse: Recovery through Exposure (SHARE) was completed by approximately half of the sample.

2 Northwest Arkansas Community Correction Center is a 120-bed minimum-security women’s prison. Women who are incarcerated there have generally been convicted of at least one non-violent felony (e.g., drug crimes, financial crimes). Duration of incarceration generally ranges from about 6 months to 3 years. The Center is licensed by the State of Arkansas as a substance use treatment facility and thus there are more therapeutic programs offered by the facility than at many prisons. Individual diagnosis of a substance use disorder is not required for women to be incarcerated there.

3 Themes are not mutually exclusive and thus percentages add up to more than 100%.

## References

[CR1] Abbott P, Magin P, Davison J, Hu W (2017). Medical homelessness and candidacy: Women transiting between prison and community health care. International Journal for Equity in Health.

[CR2] Ahmed R, Angel C, Martel R, Pyne D, Keenan L (2016). Access to healthcare services during incarceration among female inmates. International Journal of Prisoner Health.

[CR3] Alves J, Maia Â, Teixeira F (2016). Health conditions prior to imprisonment and the impact of prison on health: Views of detained women. Qualitative Health Research.

[CR4] Baker B (2019). Perinatal outcomes of incarcerated pregnant women: An integrative review. Journal of Correctional Health Care.

[CR5] Barry LC, Adams KB, Zaugg D, Noujaim D (2019). Health care needs of older women prisoners: Perspectives of the health care workers who care for them. Journal of Women & Aging.

[CR6] Besney JD, Angel C, Pyne D, Martell R, Keenan L, Ahmed R (2018). Addressing women’s unmet health care needs in a Canadian remand center: Catalyst for improved health?. Journal of Correctional Health Care.

[CR7] Biancarelli DL, Biello KB, Childs E, Drainoni M, Salhaney P, Edeza A, Mimiaga MJ, Saitz R, Bazzi AR (2019). Strategies used by people who inject drugs to avoid stigma in healthcare settings. Drug and Alcohol Dependence.

[CR8] Binswanger IA, Krueger PM, Steiner JF (2009). Prevalence of chronic medical conditions among jail and prison inmates in the USA compared with the general population. Journal of Epidemiology and Community Health.

[CR9] Binswanger IA, Stern MF, Deyo RA, Heagerty PJ, Cheadle A, Elmore JG, Koepsell TD (2007). Release from prison—A high risk of death for former inmates. The New England Journal of Medicine.

[CR10] Bucerius S, Haggerty KD, Dunford DT (2021). Prison as temporary refuge: Amplifying the voices of women detained in prison. The British Journal of Criminology.

[CR11] Calcaterra SL, Beaty B, Mueller SR, Min S-J, Binswanger IA (2014). The association between social stressors and drug use/hazardous drinking among former prison inmates. Journal of Substance Abuse Treatment.

[CR12] Carson, E. A. (2020). Bureau of Justice Statistics (BJS)—Prisoners in 2019. https://www.bjs.gov/index.cfm?ty=pbdetail&iid=7106

[CR13] Chamberlain A, Nyamu S, Aminawung J, Wang EA, Shavit S, Fox AD (2019). Illicit substance use after release from prison among formerly incarcerated primary care patients: A cross-sectional study. Addiction Science & Clinical Practice.

[CR14] Clark CB, Perkins A, McCullumsmith CB, Islam MA, Hanover EE, Cropsey KL (2012). Characteristics of victims of sexual abuse by gender and race in a community corrections population. Journal of Interpersonal Violence.

[CR15] Colbert AM, Goshin LS, Durand V, Zoucha R, Sekula LK (2016). Women in transition: Experiences of health and health care for recently incarcerated women living in community corrections facilities. Research in Nursing & Health.

[CR16] Colbert AM, Sekula LK, Zoucha R, Cohen SM (2013). Health care needs of women immediately post-incarceration: A mixed methods study. Public Health Nursing (Boston, Mass.).

[CR17] DeHart DD (2008). Pathways to prison: Impact of victimization in the lives of incarcerated women. Violence Against Women.

[CR18] Dickson MF, Staton M, Tillson M, Leukefeld C, Webster JM, Oser CB (2018). The affordable care act and changes in insurance coverage and source of health care among high-risk rural, substance-using, female offenders transitioning to the community. Journal of Health Care for the Poor and Underserved.

[CR19] Dworkin ER (2018). Risk for mental disorders associated with sexual assault: A meta-analysis. Trauma, Violence & Abuse..

[CR20] Dworkin ER, Menon SV, Bystrynski J, Allen NE (2017). Sexual assault victimization and psychopathology: A review and meta-analysis. Clinical Psychology Review.

[CR21] Fazel S, Bains P, Doll H (2006). Substance abuse and dependence in prisoners: A systematic review. Addiction.

[CR22] Fazel S, Yoon IA, Hayes AJ (2017). Substance use disorders in prisoners: An updated systematic review and meta-regression analysis in recently incarcerated men and women. Addiction.

[CR23] Fox AD, Anderson MR, Bartlett G, Valverde J, Starrels JL, Cunningham CO (2014). Health outcomes and retention in care following release from prison for patients of an urban post-incarceration transitions clinic. Journal of Health Care for the Poor and Underserved.

[CR24] Fuentes CM (2014). Nobody’s child: The role of trauma and interpersonal violence in women’s pathways to incarceration and resultant service needs. Medical Anthropology Quarterly.

[CR25] Harner H, Burgess AW (2011). Using a trauma-informed framework to care for incarcerated women. Journal of Obstetric, Gynecologic, & Neonatal Nursing: Clinical Scholarship for the Care of Women, Childbearing Families, & Newborns.

[CR26] Harner HM, Riley S (2013). The impact of incarceration on women’s mental health: Responses from women in a maximum-security prison. Qualitative Health Research.

[CR27] Hayes MO (2015). The life pattern of incarcerated women: The complex and interwoven lives of trauma, mental illness, and substance abuse. Journal of Forensic Nursing.

[CR28] Howell BA, Wang EA, Winkelman TNA (2019). Mental health treatment among individuals involved in the criminal justice system after implementation of the affordable care act. Psychiatric Services.

[CR29] Irish L, Kobayashi I, Delahanty DL (2010). Long-term physical health consequences of childhood sexual abuse: A meta-analytic review. Journal of Pediatric Psychology.

[CR30] Johnson JE, Schonbrun YC, Nargiso JE, Kuo CC, Shefner RT, Williams CA, Zlotnick C (2013). “I know if I drink I won’t feel anything”: Substance use relapse among depressed women leaving prison. International Journal of Prisoner Health.

[CR31] Karlsson ME, Zielinski MJ (2020). Sexual victimization and mental illness prevalence rates among incarcerated women: A literature review. Trauma, Violence, & Abuse.

[CR32] Knittel AK, Shook-Sa BE, Rudolph JE, Edmonds A, Ramirez C, Cohen MH, Adedimeji A, Taylor TN, Michel KG, Milam J, Cohen J, Donohue JD, Foster A, Fischl M, Konkle-Parker D, Adimora AA (2021). Incidence and prevalence of incarceration in a longitudinal cohort of women at risk for human immunodeficiency virus in the United States, 2007–2017. Journal of Women’s Health.

[CR33] Komarovskaya IA, Loper AB, Warren J, Jackson S (2011). Exploring gender differences in trauma exposure and the emergence of symptoms of PTSD among incarcerated men and women. Journal of Forensic Psychiatry & Psychology.

[CR34] Lane J, Fox KA (2013). Fear of property, violent, and gang crime: Examining the shadow of sexual assault thesis among male and female offenders. Criminal Justice and Behavior.

[CR35] LeBel TP (2012). “If one doesn’t get you another one will”: Formerly incarcerated persons’ perceptions of discrimination. The Prison Journal.

[CR36] Marlow E, White MC, Chesla CA (2010). Barriers and facilitators: Parolees’ perceptions of community health care. Journal of Correctional Health Care: The Official Journal of the National Commission on Correctional Health Care.

[CR37] Minor-Harper, S. (1982). Prisoners in state and Federal Institutions on December 31, 1980 (p. 55). Bureau of Justice Statistics. https://www.bjs.gov/content/pub/pdf/psfi80.pdf

[CR38] Muncan B, Walters SM, Ezell J, Ompad DC (2020). “They look at us like junkies”: Influences of drug use stigma on the healthcare engagement of people who inject drugs in New York City. Harm Reduction Journal.

[CR39] Nijhawan AE, Salloway R, Nunn AS, Poshkus M, Clarke JG (2010). Preventive healthcare for underserved women: Results of a prison survey. Journal of Women’s Health.

[CR40] Oser CB, Bunting AM, Pullen E, Stevens-Watkins D (2016). African american female offender’s use of alternative and traditional health services after re-entry: Examining the behavioral model for vulnerable populations. Journal of Health Care for the Poor and Underserved.

[CR41] Reeves EA, Humphreys JC (2018). Describing the healthcare experiences and strategies of women survivors of violence. Journal of Clinical Nursing.

[CR42] Reviere R, Young VD (2004). Aging behind bars: Health care for older female inmates. Journal of Women & Aging.

[CR43] Roos LE, Afifi TO, Martin CG, Pietrzak RH, Tsai J, Sareen J (2016). Linking typologies of childhood adversity to adult incarceration: Findings from a nationally representative sample. The American Journal of Orthopsychiatry.

[CR44] Sawyer, W. (2018). The Gender Divide: Tracking women’s state prison growth. https://www.prisonpolicy.org/reports/women_overtime.html

[CR45] Shavit S, Aminawung JA, Birnbaum N, Greenberg S, Berthold T, Fishman A, Busch SH, Wang EA (2017). Transitions clinic network: Challenges and lessons in primary care for people released from prison. Health Affairs (Project Hope).

[CR46] Staton M, Leukefeld C, Webster JM (2003). Substance use, health, and mental health: Problems and service utilization among incarcerated women. International Journal of Offender Therapy and Comparative Criminology.

[CR47] Substance Abuse and Mental Health Services Administration (2017). Guidelines for successful transition of people with mental or substance use disorder from jail and prison: Implementation guide. https://store.samhsa.gov/sites/default/files/d7/priv/sma16-4998.pdf

[CR48] Substance Abuse and Mental Health Services Administration (2020). After incarceration: A guide to helping women reenter the community. https://store.samhsa.gov/sites/default/files/d7/priv/sma16-4998.pdf

[CR49] Sufrin C, Beal L, Clarke J, Jones R, Mosher WD (2019). Pregnancy outcomes in US prisons, 2016–2017. American Journal of Public Health.

[CR50] Sundler AJ, Lindberg E, Nilsson C, Palmér L (2019). Qualitative thematic analysis based on descriptive phenomenology. Nursing Open.

[CR51] Vail WL, Niyogi A, Henderson N, Wennerstrom A (2017). Bringing it all back home: Understanding the medical difficulties encountered by newly released prisoners in New Orleans, Louisiana - a qualitative study. Health & Social Care in the Community.

[CR52] van Olphen, J., Eliason, M. J., Freudenberg, N., & Barnes, M. (2009). Nowhere to go: How stigma limits the options of female drug users after release from jail. *Substance Abuse Treatment, Prevention, and Policy*, *4*(1). 10.1186/1747-597X-4-10. 10.1186/1747-597X-4-10 PMC2685368 19426474

[CR53] Wang EA, Hong CS, Samuels L, Shavit S, Sanders R, Kushel M (2010). Transitions clinic: Creating a community-based model of health care for recently released California prisoners. Public Health Reports (Washington, D.C.: 1974).

[CR54] Young (2000). Women’s perceptions of health care in prison. Health Care for Women International.

[CR55] Young VD, Reviere R (2001). Meeting the health care needs of the new woman inmate: A national survey of prison practices. Journal of Offender Rehabilitation.

[CR56] Zielinski, M. J., Karlsson, M. E., & Bridges, A. J. (2021). “I’m not alone, my story matters:” Re-evaluating assumptions about conducting imaginal exposure to sexual violence memories in psychotherapy groups for incarcerated women. *Health & Justice, 9*, 25. 10.1186/s40352-021-00148-4. 10.1186/s40352-021-00148-4 PMC8482612 34591180

